# White Coats at a Crossroads: Hygiene, Infection Risk, and Patient Trust in Healthcare Attire—An Umbrella Review with Quantitative Synthesis and Stress, Weaknesses, Opportunities, and Threats Analysis

**DOI:** 10.3390/microorganisms12122659

**Published:** 2024-12-21

**Authors:** Christos Tsagkaris, Matthias Rueger, Samuel B. Tschudi, Thomas Dreher

**Affiliations:** 1 Pediatric Orthopedic and Trauma Surgery, Children’s University Hospital Zürich, 8008 Zürich, Switzerland; matthias.rueger@kispi.uzh.ch (M.R.); samuel.tschudi@kispi.uzh.ch (S.B.T.); thomas.dreher@kispi.uzh.ch (T.D.); 2 Pediatric Orthopedics, Balgrist University Hospital, University of Zurich, 8008 Zürich, Switzerland

**Keywords:** doctors, attire, contamination, pathogens, infection control, healthcare

## Abstract

White coats, traditionally symbols of physicians’ hygiene and professionalism, are now scrutinized for potential infection risks during patient interactions. This review investigates whether wearing white coats is linked to microbial contamination, infection transmission, and patient expectations. An umbrella review of peer-reviewed studies and guidelines was conducted, with searches in PubMed/Medline and Scopus using terms related to medical attire, infection control, patient perceptions, and discrimination. Ten records were included, and a bibliometric analysis was performed with VOS Viewer. Bias appraisal was conducted using the JBI Bias Assessment Toolset, and a SWOT analysis was developed to support evidence-based decision-making. Findings indicate that white coats may harbor pathogens such as *Staphylococcus aureus*, Gram-positive cocci, Gram-negative rods, and MRSA. To mitigate contamination risks, it is recommended that physicians roll up coat sleeves during examinations and that the coats receive daily laundering in healthcare settings. However, evidence supporting a coatless policy is yet to be published. Patients tend to expect physicians to wear identifiable attire, like white coats or scrubs for surgeons. Recent research in this field shifts the focus from infection control to the impact of attire on patient trust and physician–patient relationships.

## 1. Introduction

The white medical coat, also known as a lab coat, is a knee-length overcoat worn by healthcare professionals, particularly doctors and physicians. The tradition of doctors wearing white coats began in the late 19th century. Prior to this, doctors typically wore black clothing, similar to clergy members. The shift to white coats occurred to symbolize purity and cleanliness in the medical profession, to distinguish trained physicians from untrained practitioners, and to align with the growing emphasis on scientific approaches in medicine. The white color was specifically chosen because it made stains easily visible, promoting cleanliness and hygiene [1,2].

Although there are variations across countries and healthcare systems with regard to the use of white coats, it is estimated that approximately 70% of hospital doctors and medical students wear white coats [3]. Some specialties, like psychiatry and pediatrics, may prefer business attire to make patients feel more at ease. Emergency room and operating room staff often wear scrubs instead of white coats. Healthcare systems such as the National Healthcare Service of the United Kingdom have collectively replaced white coats with scrubs or business attire. The length of the white coat can also vary. Short coats (hip-length) are often worn by medical students and first-year residents, while long coats (knee-length) are typically worn by senior physicians [1,4].

White coats have become a symbol of the medical profession, helping physicians to be identifiable, but also potentiating a system of cultural beliefs among the broader community. Physicians wearing white coats over business attire are perceived as more knowledgeable, trustworthy, caring, and approachable. However, some patients experience “white coat syndrome”, where the sight of a white coat causes anxiety and elevated blood pressure readings [5,6].

In 2007, the use of white coats in hospitals in England was banned, and similar motions were considered in the United States of America, but have not been enforced so far [7]. This motion was based on concerns that white coats may act as fomites contributing to the spread of nosocomial infection [3,6]. Particular elements of them such as long sleeves and pockets render them more likely to be contaminated during contact with patients and through storing and withdrawing items and portable pieces of equipment such as stethoscopes [8]. Similar concerns were voiced for a number of medical and non-medical components of the standard physicians’ attire, ranging from stethoscopes and examination devices (e.g., neurological hammers) to ties and necklaces [8].

This debate stimulated scientific research assessing health risks and broader behavioral implications related to white coats [2,6,9]. While the majority of relevant studies focus on infection and disease control, the role of the white coat as a symbol affirming the position of a physician, making physicians, particularly female ones, more easily identifiable and contributing to the development of trust from the side of the patients were also highlighted [4,10]. A growing body of primary literature on the topic was appraised through systematic reviews published over the last decade and was also incorporated in the pertinent guidelines of international and national health authorities.

In the face of recent concerns over white coats expressed in the facility of the authors, this paper investigates whether the use of white coats is associated with an increased risk of infection, negative health outcomes, and patients’ experiences.

## 2. Materials and Methods

A literature search was performed on Pubmed/Medline and Scopus from 1 January 1991 to 1 November 2024 with keywords in the nexus of white coats and medical clothing, infection and disease control, gender role, and discrimination and affirmation in healthcare with the explicit mention of surgery and pediatric surgery. A detailed search string for each of the databases is provided in Appendix A. The review protocol was registered with Open Science Foundation (OSF, https://doi.org/10.17605/OSF.IO/F3CWR). All types of articles were initially surveyed, and their authorship, title, and abstract metadata were exported for thematic visualization. Consequently, both search engines were filtered for systematic reviews and their metadata (title, authorship, abstract) were extracted and uploaded on the literature manager software Rayyan.ai (https://www.rayyan.ai/, accessed on 9 November 2024), where they were available to two of the authors for screening and inclusion.

The inclusion criteria included systematic reviews, published in English, German, or French between 1 January 1991 and 1 November 2024, addressing the use of white coats in healthcare—clinical settings in all countries and medical and surgical disciplines reported. No limitation was set with regard to the appraisal of white coats in clinical (disease control) and behavioral (trust) settings. Studies reporting on other elements of medical attire additionally to white coats were not rejected, as long as the results associated with white coats were separately reported. The definition of a study as a systematic review was based on its classification on Pubmed/Medline and Scopus. Each included study was manually controlled to ensure that it belonged to this genre. All systematic review formats (PRISMA and non-PRISMA) were eligible, given that the search extended before the publication of the PRISMA framework. Exclusion criteria included wrong study types, full text publication in languages different than English, German, or French, and the unavailability of the full text of the publication. The reference lists as well as the websites of the World Health Organization (WHO), the European Center for Disease Prevention and Control (ECDC), and the Swiss Federal Office of Public Health (FOPH) were searched for reports and guidelines related to hospital and healthcare facilities’ hygiene and physicians’ attire. Official documents issued by international and national health authorities and organizations within the above-mentioned timeline were additionally considered eligible for inclusion under the rationale that they represent guidance on the implementation of the best available evidence in the field. The same language criteria were applied. The search was conducted according to the PRISMA Statement for Umbrella literature reviews [11]. The risk of bias was appraised with the JBI Checklist for bias appraisal in systematic reviews [12].

The included peer-reviewed studies and gray literature were tabulated. A quantitative summary of the microbial contamination of white coats reported across a number of included systematic reviews was provided. Descriptive statistics, namely range, mean, median, and interquartile range (IQR), were calculated with Statology (https://www.statology.org/, accessed on 10 November 2024). Nominal values or ranges were retrieved from the included reports, mean and median were calculated for pathogens for which at least two values were given and IQR was calculated for pathogens for which at least four values were available.

### 2.1. Bibliometric Analysis

The entire findings from Pubmed/Medline and Scopus were exported as csv files, included the title, abstract, and authorship metadata, and were uploaded on VOS Viewer (Leiden, The Netherlands) to generate a bibliographic map visualization of keywords appearing in the titles and abstracts of the detected literature [13]. Three visualization panels were generated and projected keywords that appeared in at least 5 different documents. A total of 51 keywords were included the visualization after manually excluding 1 keyword without contextual relation to the topic (“record”).

### 2.2. Stress, Weaknesses, Opportunities, and Threats (SWOT) Analysis

A SWOT analysis was conducted to reiterate the findings within healthcare facilities’ administration and infection control practices [14]. This aimed to provide a comprehensive framework for understanding the practical implications of white coat use in healthcare settings, with particular emphasis on infection control and public health standards in hospitals. The analysis was informed by both peer-reviewed and gray literature sources included in the review. The results of the literature search were structured into four primary categories: 1. strengths, 2. weaknesses, 3. opportunities, and 4. threats.

## 3. Results

**Figure 1:**
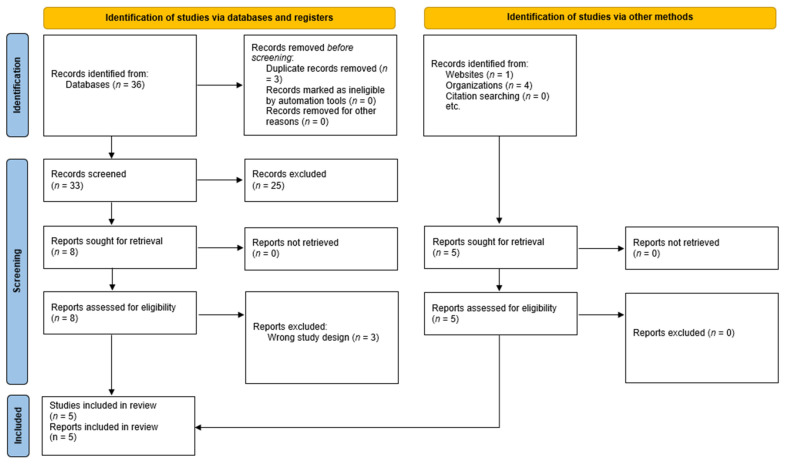
PRISMA flowchart.

A stepwise report of the search and selection of the peer-reviewed and gray literature is presented in Figure 1. Searching Pubmed/Medline for all types of publications within the defined time period revealed 152 records. Filtering for systematic reviews resulted in four results. Likewise, the search in Scopus led to 270 records, out of which 32 were labeled as systematic reviews. Three duplicate records were deleted and subsequently the titles and abstracts of 33 records were screened, revealing 8 records suitable for full text assessment. Their full texts were retrieved and five were selected for inclusion as per the inclusion and exclusion criteria. Searching the gray literature consisting of the reports and guidelines of the WHO, the ECDC, the National Institutes of Health (NIH), and the FOPH revealed five relevant records all of which were deemed suitable for inclusion. In total, 10 documents were included, tabulated into two categories (Table 1, Table 2 and Table 3) and subsequently analyzed.

The characteristics of the included peer-reviewed studies are presented in Table 1. Infection control was the focus of four systematic reviews published between 2014 and 2021, while one systematic review addressed behavioral aspects of the use of white coats and relevant patients’ perceptions. The majority of studies originated from North America and Asia, followed by Europe and Australia/New Zealand. A limited number of primary research assessed in the systematic reviews originated from Africa and South America, representing only one country from each region, namely Nigeria and Brazil. The number of studies included in each systematic review ranged from 22 to 72, depending on the range of pathogens and elements of medical equipment apart from white coats that each study assessed. The pathogens assessed included Staphylococcus, *S. aureus*, Methicillin-resistant *Staphylococcus aureus* (MRSA), Gram-positive cocci, Gram-negative rods (GNRs), *Pseudomonas aeruginosa*, Diptheroids, and vancomycin-resistant enterococci.

A quantitative summary of the pathogens identified is presented in Table 2. *S. aureus* and broadly Gram-positive cocci were most commonly detected, followed by GNRs. *S. aureus* and GNRs showed high variability, suggesting that contamination rates are quite spread out in different countries, investigations, and clinical practice settings. Skin microbiota, Pseudomonas aeruginosa and Enterobacteriaceae, were detected in 10–18% of the white coats assessed. The presence of methicillin-resistant *Staphylococcus aureus* (MRSA) reached up to 19.1% but could also be as low as 3.5% as long as isolation protocols were observed. Pathogens less commonly observed included *Streptococcus* spp. and GNRs.

White coats were found to harbor microbial contamination, including antibiotic-resistant bacteria. Uneke and colleagues (2014) observed the highest contamination rates in India and Nigeria and noted that white coats serve as potential vectors for infectious pathogens, although direct evidence of transmission to patients remains limited [15]. Haun et al. (2016) expanded on contamination sources, showing that, in addition to white coats, stethoscopes, digital devices, and neckties are also frequently contaminated with pathogens such as *S. aureus* and GNRs [8]. Goyal et al. (2019) underscored that contamination levels were not reported to differ between standard coats and coats impregnated with antimicrobial chemicals. They also discussed laundering practices with both healthcare workers and students tending to wash their coats infrequently, oftentimes less than once every 2 or every 3.5 weeks, respectively, for each group. Both groups heavily relied on home laundering [16].

Lena and colleagues (2021) focused on MRSA contamination, observing higher rates of MRSA on white coats compared to other elements of medical attire. Their findings emphasized sleeves and pockets as a principal contamination site. Adherence to MRSA isolation protocols appeared to reduce the rate of contamination. The studies surveyed in this study were inconclusive regarding the effectiveness of hospital- versus home-based laundering of white coats [17].

Finally, yet importantly, Petrilli (2014) addressed the cultural and patient perception aspects of white coat use, highlighting that formal attire, including white coats, is preferred by a significant proportion of patients, especially older individuals in Europe and Asia. Patients showed equivocal preference over this attire or scrubs in procedural specialties conducting surgical operations or endoscopic or radiological interventions. Fear associated with white coats or discriminatory behaviors against allied healthcare professionals were not noted—to the extent that patients reported their perceptions and preferences [18].

In the context of international and national (Switzerland) guidelines on hospital and primary care hygiene guidelines, only one record provides explicit guidance on white coats. In this case, their use is permitted at the wish of the institution as long as the “Bare Below the Elbows (BBE)” rule is observed and a framework for at least the weekly washing of white coats is in place [19]. Reports from the World Health Organization (WHO) highlight the need for specialized clothing provided by the healthcare facilities to provide healthcare workers in different settings of infection transmission—this focuses on personal protective equipment (PPE) and broadly includes white coats [20,21,22]. In Switzerland, along with abiding to international guidelines, the Federal Office of Public Health underscores the need for hospital-based hygiene committees and the need for regular appraisals, without taking a particular stance on the attire of healthcare professionals [23].

### 3.1. Bibliometrical Visualization

**Figure 2:**
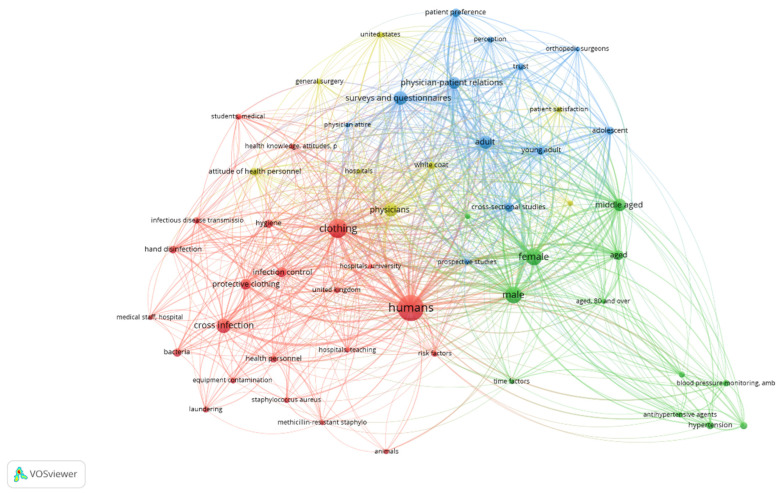
Thematic connection of keywords in research related to use of white coats. Interrelated thematic areas are marked with the same color. The shortened terms “amb” and “p” refer to “ambulatory” (blood pressure monitoring) and practices, in the frame of “health knowledge, attitudes, practices” (KAP) framework.

Figure 2 displays the connection of the keywords in the context of studies that investigated common themes, namely clothing, infection control/healthcare services, gender and physician–patient relationships.

**Figure 3:**
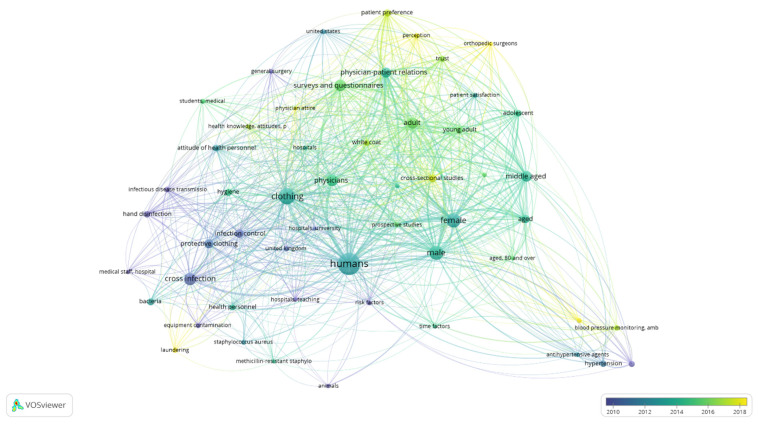
Evolution of keywords used to describe research on white coats in time. The shortened terms “amb” and “p” refer to “ambulatory” (blood pressure monitoring) and practices, in the frame of “health knowledge, attitudes, practices” (KAP) framework.

Figure 3 displays the temporal evolution of research on white coats as reflected in the keywords appearing in the published research between 2010 and 2018. Infection control as well as white coat-related hypertension prevailed in the research published between 2010 and 2012 being succeeded by a broader focus on clothing and physicians from 2012 to 2014. Research from 2014 to 2018 shifted towards doctor–patient relationships and patient preferences among elderly, adults, and young adults.

**Figure 4:**
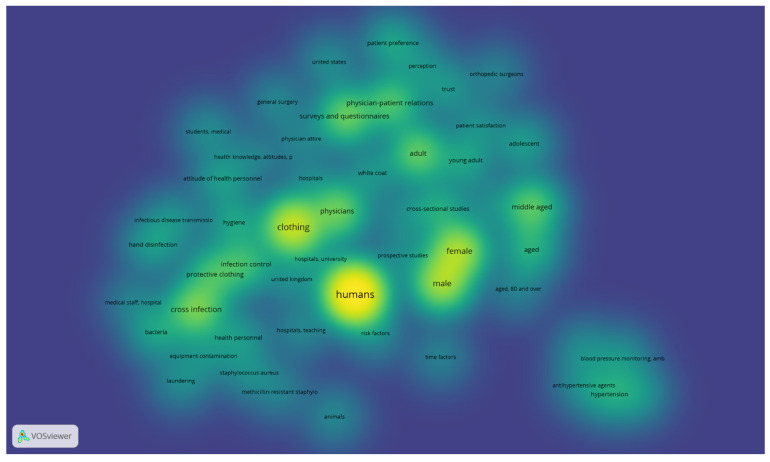
Thematic density of keywords used to describe research on white coats. Yellow marks the highest thematic density, followed by shades of green in declining intensity. The shortened terms “amb” and “p” refer to “ambulatory” (blood pressure monitoring) and practices, in the frame of “health knowledge, attitudes, practices” (KAP) framework.

Figure 4 presents the thematic density of research on white coats. Clothing, human, male/female as well as physician–patient relationships demonstrated the highest density, followed by infection control, protective clothing, and age-related terms.

### 3.2. Bias Assessment

The bias appraisal tool of the JBI Institute for Evidence Implementation (University of Adelaide, Australia) was used for bias assessment (Table 4). Two studies presented a number of concerns regarding the involvement of at least two reviewers in the selection of the reported studies. The use of a bias appraisal tool was also not explicitly reported in three studies. No studies were excluded based on this assessment, which is in line with the recommendations of the JBI Institute to provide a spherical and quantitative assessment of bias rather than nominally decide on its presence or absence based on a numerical threshold.

## 4. Discussion

Effectiveness and safety are prerequisites of healthcare delivery. Ensuring that medical attire does not pose a threat to patients’ health and wellbeing is hence pivotal. Peer-reviewed literature has investigated the use of white coats with regard to infection control and patients’ expectations. It was shown that white coats can carry infectious pathogens, including pathogens resistant to antibiotics [15,16,17]. Technical characteristics of the coats, such as the presence and retractability of long sleeves, as well as infrequent laundering in non-standardized conditions (home-based laundry) seem to aggravate the problem [16]. Nevertheless, the adherence to infection control protocols in place has shown potential in reducing the contamination of white coats, as exemplified in the case of MRSA [17]. This said, the authors could not identify evidence of spreading infections associated with the contamination of white coats by particular pathogens. Additionally, it should be noted that formal attire including a white coat is what a significant number of patients expect from physicians across the globe and particularly in Europe and Asia [18]. International and national health organizations have addressed infection control related concerns by recommending either examining patients with the sleeves of the coat retracted over the elbow or with the white coat being removed and hung at the examination space. Guidelines recommending the removal of white coats from the attire of physicians or particular components of healthcare delivery have not been detected. On the contrary, guidelines emphasize the need to provide adequate in-hospital washing infrastructure for the disinfection of healthcare workers’ attire [19,20,21,22].

While the existing literature and guidelines have provided a number of clear recommendations, such as the BBE rule and the need for regular laundering, information appears rather frugal when it comes to implementing these guidelines. Further deliberation regarding the BBE rule and the need to adapt the rules to care settings with a higher contamination and infection risk is necessary. BBE implies that no personal clothing items extend below the elbows. This recommendation can be easily implemented, if the entire medical attire is provided by the hospital (e.g., polo t-shirts). Nonetheless, ensuring that healthcare workers using private clothing items (out of choice or due to the absence of hospital clothing) abide by this rule can be challenging. When being mindful of climate parameters, it cannot be expected that all healthcare workers appear at work during winter wearing short or easily retractable sleeves compatible with the BBE rule. Additionally, healthcare workers using full skin-cover attire on cultural or religious grounds might find adhering to this rule challenging [24]. In the absence of other solutions, the use of one-use sleeves or examination aprons could ensure that contamination risk is minimized while practical and cultural norms and henceforth the wellbeing of healthcare workers is respected. It can also be argued that the BBE rule is inadequate in care settings involving highly contagious pathogens such as patients with epidemiologically important multidrug-resistant organisms (MDROs) such as MRSA, spore-forming organisms like *Clostridium difficile*, or vesicular rash associated with Herpes simplex virus (HSV) and Varicella-zoster virus (VZV) [25,26]. The use of disposable protective clothing can also be prioritized over BBE in situations involving excessive wound drainage, bodily discharges, or fecal incontinence [25,27].

Drawing the lines between the mandatory and discretionary use of disposable protective clothing can be better addressed with in-hospital appraisals of contamination and infection risk within particular departments or procedures. The need for regular, at least annual and case-specific, appraisal is however horizontal. The same applies to the daily laundering of white coats at certified (e.g., in-hospital) facilities rather than at home [19]. Establishing standards of practice (SOPs) in this regard should also take into consideration the financial cost as well as the ecological footprint of disposable items. Although the authors were not able to detect relevant cost estimations, comparisons can be drawn from the use of intermittent pneumatic compression (IPC) sleeves. Evidence from this field suggests that treating five patients with single-use sleeves creates 7 kg CO_2_eq [28]. Reprocessed sleeves seem to reduce the carbon footprint by 40%, a consideration that could be applied to support the use of reprocessed sleeves and aprons in patients’ examinations. Reviewing the standards followed in the pharmaceutical and food industries to prevent contamination could also provide insights on how clothing can serve as an effective barrier against contamination.

In a broader sphere, research on the use of white coats has timely and thematically shifted from infection control to questions addressing gender affirmation in healthcare as well as patient preference and trust between physicians and patients. The decline in the thematic representation of infection control reflects that over time a consensus on the hygienic use of white coats has been established and consequently followed, allowing white coats to be further used and appraised in the context of what their use means to patients and healthcare workers. These findings, although representing the entire literature and not only the included studies, are in line with official recommendations that do not ban, but propose a code of hygienic conduct to both healthcare workers and services, when it comes to the use of white coats.

### 4.1. SWOT Analysis

To support decision-making on the use of white coats, the authors have performed a SWOT analysis based on the reported literature (Table 5) [14]. Upholding patients’ trust and expectations speaks for maintaining the white coat as a component of the medical attire, while mitigating the risk of infection renders either the enforcement of hygienic standards or the withdrawal of white coats desirable. Permitting the use of white coats while observing patient safety (BBE) and disinfection (daily laundry) standards seems to have major potential in reducing the risks associated with white coats without compromising patients’ expectations. To live up to this expectation, mandatory daily laundering in certified facilities as well as the selective use of disposable sleeves instead of BBE in high-risk scenarios is recommended. A pragmatic approach needs to be complemented by regular appraisals of contamination and infection risk at the institutional level as well as by establishing dialog and sharing good practices with occupational safety and hygiene specialists from sectors with longtime experience in minimizing contamination risk such as microbiological, pharmaceutical laboratories, and food handling industries and facilities.

Similar solutions were prioritized in the case of stethoscopes, neckties, and name badges [29,30,31,32]. While the necessity of stethoscopes can be ranked higher than those of white coats, the same cannot be argued for neckties and name badges. Each of them makes the identification of physicians straightforward, making their interaction with patients personalized, accountable, and appropriate for most patients’ expectations. On these grounds, experience has shown that infection mitigating measures such as clips or tacking and regular disinfection are widely acceptable [19,31]. It is also reasonable to assume that the same measures are effective, given that no generalized rule to remove ties from physicians’ attire has been issued. Similar rules were implemented for covering or restricting long hair (e.g., ponytails) when in contact with patients, rather than advising healthcare workers to change their appearance for the sake of hygienic clinical practice [32]. Therefore, it becomes obvious that hygienic standards tend to become harmonized with medical attire, strengthening both infection control and peoples’ expectations.

### 4.2. Limitations and Future Research

This study is subject to a number of limitations. Although we included systematic reviews that are considered to hold the highest level of evidence, it is likely that studies not included in these reviews have not been reflected in this paper. Moreover, in the absence of relevant metanalyses as well as quantitative data sufficient for a proportion metanalysis, it was only possible to present a quantitative summary of contamination rates. The latter presents variability when taking into account the different methods and locations of sampling and microbiological examination, as well as the varied microbial epidemiology across countries and regions.

Future research needs to cover under-represented regions, especially Africa and South America, and prompt guidelines when taking into account local dressing norms. Subsequently, the effectiveness of the suggested hygienic precautions needs to be examined in operational and non-operational disciplines, and discipline-specific as well as region-specific strengths and weaknesses need to be reported. With an eye on the implementation of the proposed safety measures, the availability and cost of laundering facilities needs to be surveyed and brought to the attention of stakeholders. Beyond patients’ perspectives, healthcare workers’ perceptions with a special focus on the gender affirmative role of the white coat for female physicians need to be examined.

## 5. Conclusions

White coats can be contaminated with infectious pathogens. Examining patients with the coat’s sleeves retracted and enabling daily laundering within hospital premises is recommended to reduce contamination and subsequent infection spread risk. Patients tend to expect physicians to use a white coat or, when it comes to surgical disciplines, a type of professional attire that makes them easily identifiable. No recommendation to forbid the use of white coats has been issued at an international level. Observing hygienic precautions and ensuring that the means to implement them are available to all healthcare facilities globally is pivotal for safe healthcare practice.

## Figures and Tables

**Table 1:** Peer-reviewed literature.

Authors, Date	Geographical Allocation	Focus	Studies Included	Contamination Rate	Pertinent Comments
Uneke, 2014 [15]	Asia (India, *n* = 4); North America (*n* = 2); Africa (Nigeria, *n* = 1)	Infection control	38 (8 focusing on white coats)	All pathogens 40.7%; MRSA on 3.5–18%	Highest rates of contamination in India and Nigeria White coats can harbor potential infectious pathogens, including antibiotic-resistant bacteria Limited evidence suggesting transmission through white coats
Haun, 2016 [8]	Asia (*n* = 24), North America (*n* = 24), Europe (*n* = 18), Africa (*n* = 5) Australia (*n* = 1)	Infection control	72	MRSA contamination of 0–16%; GNR contamination of white coats ranged from 0 to 42%	Stethoscopes, digital devices, white coats, and neckties commonly contaminated with bacterial pathogens including *Staphylococcus aureus* and GNRs
Goyal, 2019 [16]	Not reported	Infection control	22 (11 focusing on white coats)	*S. aureus* contamination of 5–72% of white coats; MRSA on 4%; Enterobacteriaceae on 2–17%; *Pseudomonas aeruginosa* on 5–18%; Skin microbiota on 14%; Diptheroids on 12%; Vancomycin-resistant enterococci on 1–3%; GNRs 8%; *Streptococcus* spp. on 2%;	No conclusive data on the association of contamination with particular types of fabric Pathogen-specific survival times No difference in contamination of conventional coats and coats impregnated with antimicrobial chemicals or with silver-embedded fabric 5–65% of healthcare workers laundering their white coat ≤1 time every 2 weeks, students once every 3.5 weeks Home laundry among 4–89% of healthcare workers Provide white coat hooks in residents’ offices, conference rooms, and throughout hallways within clinical settings
Lena, 2021 [17]	North America (*n* = 11); Asia (*n* = 6); Europe (*n* = 4); South America (*n* = 1)	Infection control	23 studies, 1760 healthcare workers	MRSA on 3.5–19.1% of short and long-sleeved coats/uniforms	MRSA isolation rates higher in white coats compared to other attire High rate of sleeve contamination and subsequent spread of pathogens in pockets White coat contamination affected proportionally by adherence to MRSA isolation protocols Conflicting data on effectiveness of professional laundering is over home laundering
Petrilli, 2014 [18]	North America (*n* = 12); Europe (*n* = 10); Australia and New Zealand (*n* = 2); Asia (Middle East, *n* = 1); South America (Brazil, *n* = 1)	Behavior	30 studies, 11,533 patients	NA	Formal attire and white coats preferred in 60% of studies Preference for white coats more prevalent among older patients in Europe and Asia Limited preference for white coats over scrubs in procedural specialties

GNRs: Gram-negative rods; MRSA: Methicillin-resistant *Staphylococcus aureus*; NA: Not applicable.

**Table 2:** Quantitative summary of microbial contamination of white coats.

Pathogen	Contamination Rates (%)	Range (%)	Median (%)	Mean (%)	IQR	Study
MRSA	0–19.1	19.1	10	10.1	14.5%	Uneke, 2014 [15]; Haun, 2016 [8]; Goyal, 2019 [16]; Lena, 2021 [17]
*Staphylococcus aureus*	5–72	67	38.5	38.5	NA	Goyal, 2019 [16]
GNRs	0–42	42	22	22	NA	Haun, 2016 [8]; Goyal, 2019 [16]
*Pseudomonas aeruginosa*	5–18	13	11.5	11.5	NA	Goyal, 2019 [16]
Enterobacteriaceae	2–17	15	9.5	9.5	NA	Goyal, 2019 [16]
Vancomycin-resistant enterococci	1–3	2	1.5	1.5	NA	Goyal, 2019 [16]
Gram-positive cocci	76.5	NA	NA	NA	NA	Uneke, 2014 [15]
Diptheroids	12	NA	NA	NA	NA	Goyal, 2019 [16]
Skin microbiota	14	NA	NA	NA	NA	Goyal, 2019 [16]
*Streptococcus* spp.	2	NA	NA	NA	NA	Goyal, 2019 [16]

GNRs: Gram-negative rods; MRSA: Methicillin-resistant *Staphylococcus aureus*; IQR: interquartile range; NA: Not applicable.

**Table 3:** Official reports of international and national health organizations and agencies addressing hospital hygiene, use of white coats, and best practices to reduce risk of contamination.

Title	Date	Country	Organization	Pertinent Comments
Expert Guidance: Healthcare Personnel Attire in Non-Operating Room Settings	2014	USA	SHEA	“Bare below the elbows” (BBE) White coat washing at least once weekly or at presence of soiling Washing at hospital facilities or with special precautions at residential settings Need for white coat hooks in examination settings Need for more robust evidence
Guidelines on core components of infection prevention and control programmes at the national and acute health care facility level	2016	Global	WHO	Establishment of water and laundry systems for safe washing of hospital environments and equipment No explicit mention of white coats
MINIMUM REQUIREMENTS for infection prevention and control programmes	2019	Global	WHO	Specialized clothing or equipment worn to protect healthcare worker or any other person from infection No explicit mention of white coats
Strengthening infection prevention and control in primary care	2021	Global	WHO	Specialized clothing or equipment worn to protect healthcare worker or any other person from infection No explicit mention of white coats
Structural minimal precautions for the prevention of hospital—infections in swiss acute hospitals	2022	Switzerland	FOPH	Need to establish hygiene committees at hospital levels Need to conduct annual internal hygiene audits No explicit mention of white coats

USA: United States of America; SHEA: Society for Healthcare Epidemiology of America—Guidelines Committee; WHO: World Health Organization; FOPH: Federal Office of Public Health—Division of Communicable Diseases Infection Control and Control Measures Section.

**Table 4:** JBI bias assessment checklist.

Question	Uneke, 2014 [15]	Haun, 2016 [8]	Goyal, 2019 [16]	Lena, 2021 [17]	Petrilli, 2014 [18]
1. Is the review question clearly and explicitly stated?	Yes	Yes	Yes	Yes	Yes
2. Were the inclusion criteria appropriate for the review question?	Yes	Yes	Yes	Yes	Yes
3. Was the search strategy appropriate?	Yes	Yes	Yes	Yes	Yes
4. Were the sources and resources used to search for studies adequate?	Yes	Yes	Yes	Yes	Yes
5. Were the criteria for appraising studies appropriate?	Unclear	Yes	Yes	Yes	Yes
6. Was critical appraisal conducted by two or more reviewers independently?	Unclear	Unclear	Yes	Yes	Yes
7. Were there methods to minimize errors in data extraction?	Unclear	Unclear	Unclear	Yes	Yes
8. Were the methods used to combine studies appropriate?	Unclear	Unclear	Unclear	Yes	Yes
9. Was the likelihood of publication bias assessed?	No	No	No	Yes	Yes
10. Were recommendations for policy and/or practice supported by the reported data?	Yes	Yes	Yes	Yes	Yes
11. Were the specific directives for new research appropriate?	Yes	Yes	Yes	Yes	Yes

**Table 5:** Strengths, weaknesses, opportunities, and threats (SWOT) analysis focusing on use of white coats in healthcare provision settings and adherence to relevant infection-control protocols.

Aspect	Explanation
**Strengths**	- White coats provide professional appearance, potentially increasing patient confidence. - Allows for easy identification of healthcare professionals. - Supports the tradition of professionalism in clinical settings by meeting patients’ expectations.
**Weaknesses**	- High contamination rates across various pathogens. - Possible vector for hospital-acquired infections, undermining infection control efforts. - Varying laundering practices create inconsistency in hygiene.
**Opportunities**	- Opportunity to integrate hygienic precautions in the use of white coats (e.g., daily laundered scrubs, bare below elbows). - Enhancing staff awareness and training on hygiene practices and infection control. - Implementing hooks or coat-free zones to reduce contamination risks. - Introduce daily mandatory laundering at certified facilities. - Consider additional single-use protective equipment in high-risk settings. - Explore and share good practices with microbiological laboratories as well as with the pharmaceutical and food industries.
**Threats**	- Disappointment of healthcare staff accustomed to traditional white coats. - Patient dissatisfaction if white coats are perceived as more professional. - Potential cost implications for laundering, PPE, and alternative attire solutions.

## Data Availability

No primary data were generated within the frame of this article.

## Appendix A

PICO Statement

Is the use of white coats by healthcare professionals, compared to alternative attire, associated with an increased risk of infection transmission, adverse health outcomes, or negative patient experiences in clinical settings?

PICO components:

- Population (P): Healthcare professionals and patients in clinical settings.
- Intervention (I): Use of white coats.
- Comparison (C): Absence of white coats or alternative types of medical attire (e.g., scrubs).
- Outcome (O): Increased risk of infection transmission, negative health outcomes (e.g., contamination, pathogen spread), and negative patient experiences (e.g., discrimination, fear, stress).

Search timeline:

January 1991–November 2024

Search string:

Pubmed:

(“white coats” OR “doctor coat” OR “medical coat”) AND (“hygiene” OR “infection control” OR “contamination” OR “pathogen transmission” OR “medical errors” OR “cross-contamination” OR “infection prevention” OR “bacterial load” OR “cleaning frequency” OR “discrimination” OR “gender affirmation” OR “gender equity” OR “surgery” OR “pediatric surgery” OR “orthopedics”).

Scopus:

TITLE-ABS-KEY (“white coats” OR “doctor coat” OR “medical coat”) AND TITLE-ABS-KEY (“hygiene” OR “infection control” OR “contamination” OR “pathogen transmission” OR “medical errors” OR “cross-contamination” OR “infection prevention” OR “bacterial load” OR “cleaning frequency” OR “discrimination” OR “gender affirmation” OR “gender equity” OR “surgery” OR “pediatric surgery” OR “orthopedics”) AND PUBYEAR > 1990 AND PUBYEAR < 2025.

## References

[1] Hochberg M.S. (2007). The Doctor’s White Coat—An Historical Perspective. AMA J. Ethics.

[2] Magos A. (2024). White Coats in Hospitals. BMJ.

[3] Landry M., Dornelles A.C., Hayek G., Deichmann R.E. (2013). Patient Preferences for Doctor Attire: The White Coat’s Place in the Medical Profession. Ochsner J..

[4] Anvik T. (1990). Doctors in a White Coat-What Do Patients Think and What Do Doctors Do? 3739 Patients, 137 General Practitioners, and 150 Staff Members Give Their Answers. Scand. J. Prim. Health Care.

[5] Brase G.L., Richmond J. (2004). The White–Coat Effect: Physician Attire and Perceived Authority, Friendliness, and Attractiveness. J. Appl. Soc. Psychol..

[6] Cochran A., Upchurch G.R. (2021). Has the Physician’s White Coat Seen Its Day?. JAMA Netw. Open.

[7] (2007). Doctors’ White Coats Banned in Britain. Emerg. Med. News.

[8] Haun N., Hooper-Lane C., Safdar N. (2016). Healthcare Personnel Attire and Devices as Fomites: A Systematic Review. Infect. Control Hosp. Epidemiol..

[9] Romano M.J. (2018). White Privilege in a White Coat: How Racism Shaped My Medical Education. Ann. Fam. Med..

[10] Mishra S.K., Maharjan S., Yadav S.K., Sah N.P., Sharma S., Parajuli K., Sherchand J.B. (2020). Bacteria on Medical Professionals’ White Coats in a University Hospital. Can. J. Infect. Dis. Med. Microbiol..

[11] Rethlefsen M.L., Kirtley S., Waffenschmidt S., Ayala A.P., Moher D., Page M.J., Koffel J.B., Blunt H., Brigham T., Chang S. (2021). PRISMA-S: An Extension to the PRISMA Statement for Reporting Literature Searches in Systematic Reviews. Syst. Rev..

[12] JBI Critical Appraisal Tools|JBI. https://jbi.global/critical-appraisal-tools.

[13] Arruda H., Silva E.R., Lessa M., Proença D., Bartholo R. (2022). VOSviewer and Bibliometrix. J. Med. Libr. Assoc..

[14] Mercieca M., Schembri F., Inglott A.S., Azzopardi L.M. (2023). SWOT Analysis. Pharm. Technol..

[15] Uneke C.J. (2014). Are Non-Critical Medical Devices Potential Sources of Infections in Healthcare Facilities?. World Health Popul..

[16] Goyal S., Khot S.C., Ramachandran V., Shah K.P., Musher D.M. (2019). Bacterial Contamination of Medical Providers’ White Coats and Surgical Scrubs: A Systematic Review. Am. J. Infect. Control.

[17] Lena P., Ishak A., Karageorgos S.A., Tsioutis C. (2021). Presence of Methicillin-Resistant Staphylococcus Aureus (MRSA) on Healthcare Workers’ Attire: A Systematic Review. Trop. Med. Infect. Dis..

[18] Michael Petrilli C., Mack M., Janowitz Petrilli J., Hickner A., Saint S., Chopra V. (2015). Understanding the Role of Physician Attire on Patient Perceptions: A Systematic Review of the Literature—Targeting Attire to Improve Likelihood of Rapport (TAILOR) Investigators. BMJ Open.

[19] Bearman G., Bryant K., Leekha S., Mayer J., Munoz-Price L.S., Murthy R., Palmore T., Rupp M.E., White J. (2014). Healthcare Personnel Attire in Non-Operating-Room Settings. Infect. Control Hosp. Epidemiol..

[20] Guidelines on Core Components of Infection Prevention and Control Programmes at the National and Acute Health Care Facility Level. https://www.who.int/publications/i/item/9789241549929.

[21] World Health Organization (2019). Minimum Requirements for Infection Prevention and Control Programmes.

[22] WHO (2021). Strengthening Infection Prevention and Control in Primary Care.

[23] Sur Les Exigences Structurelles Minimales IAS—Swissnoso. https://www.swissnoso.ch/fr/recherche-developpement/strukturelle-mindestanforderungen-hai/ueber-die-strukturellen-mindestanforderungen.

[24] Szumska E., Czajkowski P., Zablocki M., Rozkiewicz D. (2023). A Multifaceted Approach to the “Bare below the Elbow” Concept and Hand Hygiene Compliance among Healthcare Professionals—Multicenter Population-Based Study. Int. J. Environ. Res. Public Health.

[25] Douedi S., Douedi H. (2023). Precautions, Bloodborne, Contact, and Droplet. StatPearls.

[26] NHS England Chapter 1: Standard Infection Control Precautions (SICPs). https://www.england.nhs.uk/national-infection-prevention-and-control-manual-nipcm-for-england/chapter-1-standard-infection-control-precautions-sicps/.

[27] Gottenborg E.W., Barron M.A. (2015). Isolation Precautions in the Inpatient Setting. Hosp. Med. Clin..

[28] Lichtnegger S., Meissner M., Paolini F., Veloz A., Saunders R. (2023). Comparative Life Cycle Assessment Between Single-Use and Reprocessed IPC Sleeves. Risk Manag. Healthc. Policy.

[29] Stephens B., Azimi P., Thoemmes M.S., Heidarinejad M., Allen J.G., Gilbert J.A. (2019). Microbial Exchange via Fomites and Implications for Human Health. Curr. Pollut. Rep..

[30] Campos-Murguía A., León-Lara X., Muñoz J.M., Macías A.E., Álvarez J.A. (2014). Stethoscopes as Potential Intrahospital Carriers of Pathogenic Microorganisms. Am. J. Infect. Control.

[31] Pace-Asciak P., Bhimrao S.K., Kozak F.K., Westerberg B.D. (2018). Health Care Professionals’ Neckties as a Source of Transmission of Bacteria to Patients: A Systematic Review. CMAJ Open.

[32] Jakhar D., Kaur I., Kandhari R., Kaul S., Garg P., Bansal S. (2020). Hair Care during COVID-19: Practical Tips for Health Care Workers. Indian J. Med. Sci..

